# Melatonin-inspired hybrid spheroids accelerate intestinal repair via YAP-driven fetal reprogramming

**DOI:** 10.1016/j.ajps.2026.101176

**Published:** 2026-06-20

**Authors:** Yoojin Seo, Ji-Su Ahn, Nhu-Nam Nguyen, Hyeon Seo Lee, Yunji Lee, Seong Hui Kim, Jeong Hyun Yu, Ji-Won Yang, Hee-Jeong Park, Hansong Lee, Tae-Hoon Shin, Byung-Chul Lee, Eui-Suk Sung, Jung-Hwan Lee, Won Kyu Kim, Jung-Min Oh, Dongjun Lee, Yun Hak Kim, Jee-Heon Jeong, Hyung-Sik Kim

**Affiliations:** a Department of Oral Biochemistry, Dental and Life Science Institute, School of Dentistry, Pusan National University, Yangsan 50612, South Korea; b Department of Precision Medicine, School of Medicine, Sungkyunkwan University, Suwon 16419, South Korea; c Department of Life Science in Dentistry, School of Dentistry, Pusan National University, Yangsan 50612, South Korea; d Education and Research Team for Life Science on Dentistry, Pusan National University, Yangsan 50612, South Korea; e Medical Research Institute, Pusan National University, Yangsan 50612, South Korea; f Department of Laboratory Animal Medicine, College of Veterinary Medicine and Veterinary Medical Research Institute, Jeju National University, Jeju 63243, South Korea; g Division of Biological Sciences, Sookmyung Women’s University, Seoul 04310, South Korea; h Department of Otorhinolaryngology-Head and Neck Surgery, Biomedical Research Institute, Pusan National University School of Medicine, Yangsan Pusan National University Hospital, Yangsan 50612, South Korea; i Department of Biomaterials Science, College of Dentistry, Dankook University, Cheonan 31116, South Korea; j Department of Convergence Medicine, Yonsei University Wonju College of Medicine, Wonju 26426, South Korea; k Natural Products Research Center, Korea Institute of Science and Technology, Gangneung 25451, South Korea; l Department of Convergence Medicine, Pusan National University School of Medicine, Yangsan 50612, South Korea; m Department of Anatomy, Pusan National University School of Medicine, Yangsan 50612, South Korea

**Keywords:** Hybrid spheroid, Melatonin, Mesenchymal stem cell, Prostaglandin E2, Fetal reprogramming, Inflammatory bowel disease

## Abstract

Following injury, prostaglandin E_2_ (PGE_2_) drives intestinal epithelial repair by inducing revival stem cells (RSCs), which compensate for the loss of homeostatic Lgr5^+^ stem cells. Using intestinal organoid models, we demonstrate that melatonin potentiates the PGE_2_- or damage-induced RSC emergence by rewiring cellular plasticity toward a fetal-like state and sustaining pro-regenerative YAP activity, thereby enhancing overall repair capacity. To translate this finding into a therapeutic application, we developed a biohybrid heterospheroid (Mel-HS) by combining melatonin-loaded poly(lactic-co-glycolic acid) microspheres with 3D-cultured mesenchymal stem cells (MSCs), which serve as a PGE_2_ source. We confirmed that this biohybrid construct preserves the paracrine capacity of MSCs to secrete PGE_2_. Notably, Mel-HS demonstrates superior *in vivo* retention compared with naive 3D-MSCs, underscoring the cytoprotective effect of encapsulated melatonin in enhancing MSC viability. Furthermore, Mel-HS promoted robust RSC induction while simultaneously providing protection against inflammatory- and oxidative insults *in vitro*. In a colitis model, Mel-HS accelerated mucosal healing through the dual mechanisms—immunomodulation and enhanced RSC-driven repair—resulting in marked clinical improvement. Collectively, our findings highlight the therapeutic potential of enhancing endogenous regeneration with melatonin and MSCs, establishing a promising framework for next-generation biohybrid cell therapeutics in inflammatory bowel disease management.

## Graphical abstract

**Figure:**
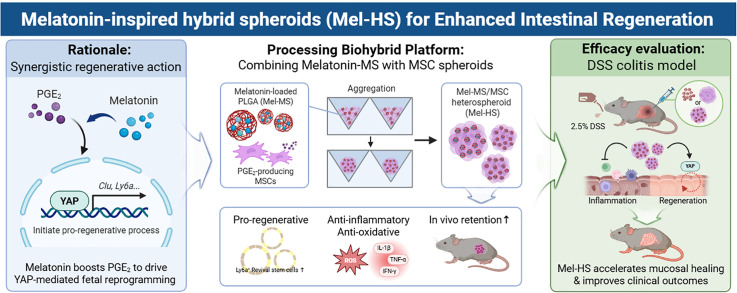

## 1 Introduction

Inflammatory bowel disease (IBD) encompasses chronic conditions marked by recurrent gastrointestinal inflammation [1]. Current therapeutic strategies for IBD focus on achieving and maintaining remission, primarily through anti-inflammatory agents, immunosuppressants, and targeted biologics that modulate the immune response [2,3]. In addition, considering that IBD is associated with a significant impairment of the intestinal barrier, enhancing endogenous regenerative capacity may be crucial for restoring epithelial integrity and function. Therefore, integrating regenerative approaches with conventional immunotherapies can accelerate recovery and improve patient outcomes in IBD management.

Crypt base columnar cells (CBCs) function as homeostatic intestinal stem cells (ISCs) essential for epithelial renewal [4,5]. Following injury-induced depletion of CBCs, intestinal regeneration is driven by multiple cell types that undergo reprogramming to a fetal-like state, followed by redifferentiation [[6], [7], [8], [9]]. Accumulating evidence indicates that the emergence of clusterin (Clu)- and stem cell antigen-1 (Sca-1)-positive cells, often referred to as revival stem cells (RSCs), is a prerequisite for epithelial repair [10]. The activation of RSCs is fine-tuned by diverse signals—ranging from stromal niche factors such as prostaglandin E_2_ (PGE_2_) to immune modulators and even pathogen-derived factors—that enhance their emergence in a context-dependent manner [11,12]. In particular, we recently reported that melatonin potentiated PGE_2_-induced RSC generation both *in vitro* and *in vivo* and promoted improved recovery from experimental colitis [13]. Mechanistically, melatonin alone does not directly induce RSC formation but instead enhances epithelial stemness, thereby generating an “intermediate-like” state poised for RSC induction upon PGE_2_ exposure. These findings reveal a novel role for intestinal melatonin in epithelial repair and expand its known functions beyond the regulation of intestinal motility and immunity [14,15].

Given this background, the present study leverages the synergy between melatonin and PGE_2_ to enhance the endogenous reparative capacity of the intestinal epithelium. PGE_2_ is a master regulator of gut repair and plays a pivotal role in promoting RSC generation; however, direct stimulation of endogenous PGE_2_ signaling to initiate regenerative responses presents significant challenges and carries potential risks, given its involvement in inflammation and tumor progression [11]. To overcome these limitations and rapid degradation of directly administered PGE_2_, we utilized mesenchymal stem cells (MSCs) as a sustained biological source of PGE_2_ [[16], [17], [18], [19], [20]]. We generated MSC spheroids (3D-MSCs) and combined them with melatonin-loaded poly (lactic-co-glycolic acid) (PLGA) particles for sustained delivery, thereby creating melatonin-loaded MSC heterospheroids (Mel-HS). Employing both *in vitro* intestinal organoid (IO) system and *in vivo* dextran sulfate sodium (DSS)-induced colitis model, our data demonstrate that Mel-HS enhances endogenous repair capacity by boosting reprogramming of injured epithelium and activating RSC populations, which are crucial for intestinal reconstitution in the absence of homeostatic CBCs.

## 2 Materials and methods

### 2.1 Materials

FUCCI lentiviral vectors (Addgene plasmid #86849) and the p27-mVenus reporter (Addgene plasmid #176651) were gifts from Kevin Brindle & Duncan Jodrell and Mohamed Bentires-Alj, respectively. 2-Iodomelatonin (2I), 8-M-PDOT (8M), melatonin, PGE_2_ and 5-fluorouracil (5-FU) were purchased from MedChemExpress (NJ, USA). Tert‑butyl hydroperoxide, PLGA, and polyvinyl alcohol (PVA) were purchased from Sigma-Aldrich (MO, USA). Recombinant IFN-γ, IL-1β, and TNF-α were obtained from PeproTech (NJ, USA). Dichloromethane was purchased from Junsei Chemical (Japan). Human umbilical cord blood-derived MSCs (UCB-MSCs) were obtained from Kangstem Biotech (South Korea), while human tonsil-derived MSCs (T-MSCs) were provided by Dr. B.J. Lee (Pusan National University Hospital, South Korea). MEM-α, fetal bovine serum (FBS), penicillin/streptomycin (P/S), and TrypLE were obtained from Gibco (NY, USA). AggreWell™400 was purchased from STEMCELL Technologies (Canada). DSS was purchased from MP Biomedicals (OH, USA). C57BL/6 male mice were purchased from Orient Bio (South Korea). Click-iT EdU Flow Cytometry Assay Kit, SYTOX reagent, CellROX Reagent, and Hoechst 33342 were obtained from Thermo Fisher Scientific (MA, USA). The MPO Activity Assay Kit was purchased from Abcam (MA, USA). Total RNA was extracted using the Qiagen RNeasy Mini Kit (Germany). SYBR Green reagent and PRO-PREP lysis buffer were obtained from iNtRON Biotechnology (South Korea). Primary antibodies were purchased from ABclonal (Arx, Epcam, Lysozyme, Muc2; MA, USA), Cell Signaling Technology (cleaved caspase-3, E-cadherin, non-phosphorylated YAP, phosphorylated YAP, total YAP; MA, USA), Proteintech (GAPDH; IL, USA), Thermo Fisher Scientific (Ki-67, Reg3β), and Abcam (Olfm4). Ly6a-FITC and Ly6a-PE antibodies were purchased from BioLegend (CA, USA). Fc receptor blocking reagent was purchased from Miltenyi Biotec (Germany). CD45-PE, CD31-PE, TER-119-PE, and CD326-APC antibodies were obtained from BD Biosciences (CA, USA). OCT compound was purchased from Sakura Finetek (Japan).

### 2.2 Organoid culture and image-based analysis

Mouse IOs were established from small intestinal crypts, following a previously published protocol [16]. To monitor cell cycle dynamics and p27 expression, IOs were transduced with lentiviral vectors for the FUCCI reporters or a p27-mVenus reporter [21]. Organoids were treated with 2I (200 µM), 8 M (200 µM), melatonin (500 µM), PGE_2_ (500 nM), 5-FU (5 µM), tert‑butyl hydroperoxide (250 µM), IFN-γ (2.5 ng/ml), IL-1β (2.5 ng/ml), or TNF-α (2.5 ng/ml), as indicated in each experiment. Organoid images were captured with BioTek Cytation 5 Cell Imaging Multi-Mode Reader (Agilent, Santa Clara, *CA*) and processed with Gen5 software using an image analysis module.

### 2.3 Gene chip microarray analysis and gene set enrichment analysis (GSEA)

Three independent organoid lines were prepared for both the control group and the melatonin-treated group. Microarray analysis was conducted by Macrogen (South Korea). The GSEA tool (v4.2.2) was used to perform functional enrichment analysis of the transcriptome. Gene sets were obtained from the Molecular Signatures Database (Hallmark collection) of the Broad Institute and from published studies, as detailed in Data Set 1.

### 2.4 Preprocessing and analysis of scRNA-seq data

The single-cell RNA sequencing (scRNA-seq) dataset of mouse IOs treated with melatonin was obtained from the Gene Expression Omnibus database (accession number GSE293340). Initial processing and quality control were performed using the Seurat R package [22]. Following filtering, the data were log-normalized using a scale factor of 10,000, and variable features essential for cell type specification were identified using the variance-stabilizing transformation method. Principal component analysis (PCA) was then conducted on the preprocessed scRNA-seq data. The first 15 principal components were selected and subsequently used for dimensionality reduction with the t-distributed stochastic neighbor embedding (t-SNE) algorithm. To specifically isolate enteroendocrine (EEC) clusters, established canonical markers, including “Chgb”, “Chga”, “Cck”, “Neurog3” and “Sct” were used to identify clusters with high expression of these genes. For a more focused analysis of heterogeneity within the EEC population, the clusters identified as EECs were extracted from the main dataset and underwent a reprocessing pipeline tailored for high-resolution subclustering. Subsequently, density-based spatial clustering of applications with noise was applied to the PCA-reduced space of the EEC subset, successfully resolving the population into four distinct EEC subclusters.

### 2.5 Fabrication and characterization of melatonin-loaded microspheres (Mel-MS)

Mel-MS were synthesized using the emulsification solvent-evaporation method. Briefly, a mixture containing PLGA (90 mg) and melatonin (10 mg) dissolved in dichloromethane was added dropwise to 5 ml of a 1% (w/v) PVA solution. The mixture was emulsified for 5 min at 13,000 rpm using a homogenizer to generate an oil-in-water (O/W) emulsion. The emulsion was subsequently transferred to 60 ml of 1% (w/v) PVA solution (aqueous phase) and stirred at room temperature for 5 h to allow evaporation of the organic solvent. The resulting microspheres were collected by centrifugation, washed and freeze-dried, and then characterized using scanning electron microscopy (SEM) (JSM-IT 800, JEOL). Microspheres were randomly selected and their size distributions were determined using Image J software. The drug loading capacity (LC) and encapsulation efficiency (EE) of Mel-MS were determined by high-performance liquid chromatography (HPLC) using a C_18_ column (5 µm, 150 × 4.6 mm). The mobile phase consisted of acetonitrile and water (70:30, v/v) delivered at a flow rate of 1 mL/min in isocratic mode, with detection at 220 nm using a PDA detector.

### 2.6 Generation of Mel-MS/MSC heterospheroids (Mel-HS)

UCB-MSCs and T-MSCs were cultured in MEM-α supplemented with 10% FBS and 1% P/S. 3D-MSCs and Mel-HS were generated using AggreWell™400 incorporating 1000 cells per spheroid with or without the addition of 0.7 µg Mel-MS, respectively.

### 2.7 Mouse models for DSS-induced colitis and evaluation

The animal experiments were approved by the Institutional Animal Care and Use Committee of Pusan National University (L2023–022-A1C0). All experiments were performed using 8–10-week-old C57BL/6 male mice maintained under specific pathogen-free conditions. To induce experimental colitis, 2.5% DSS (w/v) was administered in the drinking water for 5 d. The control group (*n* = 10) received regular drinking water. The DSS-treated mice were divided into six groups: PBS (*n* = 20), Mel-MS (MS; *n* = 16), 3D-MSCs derived from UCB (3DU; *n* = 21) or tonsil (T) (3DT; *n* = 10), and Mel-HS containing UCB-MSCs (HSU; *n* = 20) or T-MSCs (HST; *n* = 10). A total of 2 × 10^3^ of 3D-MSCs or Mel-HS, or 1.4 mg Mel-MS in 200 µl of PBS, were injected intraperitoneally on either Day 1 (to assess preventive effects) or Day 5 (to evaluate therapeutic effects). Body weight and survival rate were monitored daily. In accordance with ethical guidelines, mice showing a body weight reduction greater than 30% were euthanized. The disease activity index (DAI) was determined on Day 10 based on the average scores of weight loss (0–4), stool consistency (0–4), rectal bleeding (0–4), coat roughness (0–4) and general activity (0–2), after which mice were euthanized using inhalational anesthesia. To label proliferating cells, EdU (50 mg/kg) was administered intraperitoneally 24 h before sacrifice. To assess disease severity, epithelial disruption and immune cell infiltration were quantified in H&E-stained samples. In addition, the number of goblet cells in the villi was quantified with AB-PAS-stained tissues to evaluate mucosal healing and protection.

### 2.8 RNA extraction and real-time qPCR

Total RNA was extracted using the RNeasy Mini Kit. Real-time quantitative PCR (qPCR) was performed using the ABI 7500 Real-Time PCR System (Applied Biosystems, CA, USA) with SYBR Green reagent. The mRNA expression levels of target genes were normalized to *Gapdh*. The primer sequences used in this study are listed in Table 1.

**Table 1:** Primer sequences utilized in this study.

Gene	Forward primer	Reverse primer
*Clu*	GCTGCTGATCTGGGACAATG	ACCTACTCCCTTGAGTGGACA
*Gapdh*	GGAAGGGCTCATGACCAC	GCAGGGATGATGTTCTGG
*Lgr5*	GGGAGCGTTCACGGGCCTTC	GGTTGGCATCTAGGCGCAGGG
*Ly6a*	GAAAGAGCTCAGGGACTGGAGTGTT	TTAGGAGGGCAGATGGGTAAGCAA
*Olfm4*	ATTCGCTATGGCCAAGGAGG	GAGGGGCCGATTCACATCAA

### 2.9 Protein extraction and western blot

Samples were harvested and lysed in PRO-PREP lysis buffer and standard Western blotting was performed with primary antibodies described in Section 2.1, each at 1:1000 except GAPDH (1:50,000).

### 2.10 Flow cytometry (FC) analysis

For FC analysis of organoids, organoids were dissociated with TrypLE at 37 °C for 5 min and incubated with antibodies including *Ly6a*/Sca1-FITC or *Ly6a*/Sca1-PE for 1 h at 4 °C. For proliferation analysis, organoids were pulsed for 2 h with 10 µM EdU. Following incubation, organoids were dissociated into a single-cell suspension, and EdU incorporation was quantified by FC using the Click-iT EdU FC Assay Kit. To detect cellular viability or intracellular reactive oxygen species (ROS) levels, organoids were incubated with either SYTOX reagent or CellROX Reagent for 30 min at 37 °C and then subjected to FC. For *in vivo* analysis, approximately 5 cm of ileum tissue was dissociated at 37 °C in a buffer containing 10 mM EDTA and 3% FBS in PBS to obtain a single-cell suspension. The isolated cells were first incubated with an Fc receptor blocking reagent. Subsequently, cells were stained at 4 °C for 1 h with the following antibodies: Ly6a-FITC, CD45-PE, CD31-PE, TER-119-PE and CD326-APC. Following a final wash step, samples were acquired and analyzed using a BD Accuri™ C6 Plus flow cytometer (BD Biosciences).

### 2.11 Immunohistochemistry and immunofluorescence

For preparation of frozen section, harvested intestinal tissues or organoids were fixed in 4% paraformaldehyde and immersed in 30% sucrose and then embedded in an OCT compound. Sections (10 µm) were subjected to conventional immunohistochemical staining with the primary antibodies described in Section 2.1, each used at a dilution of 1:1000. For the quantification of Olfm4 intensity (Fig. 6G), stained spots exceeding the designated threshold value (relative expression over 20,000) were identified, and their areas were calculated using a BioTek Cytation 5 Cell Imaging Multi-Mode Reader. Histological analysis of PAS and H&E staining was conducted as previously described [23].

### 2.12 Statistical analysis

Results are presented as the mean ± SEM, derived from at least three independent experiments. Statistical significance was determined using one-way ANOVA or unpaired two-tailed Student’s *t*-test, with *P* < 0.05 considered statistically significant. To adjust for multiple comparisons and minimize the risk of Type I error, post-hoc corrections using the Bonferroni or Tukey methods were applied where appropriate. All statistical analyses were performed using GraphPad Prism 9 software. For scRNA-seq data, the Wilcoxon rank sum test in R was used to assess statistical significance between groups.

## 3 Results and discussion

### 3.1 Melatonin induces a quiescent-like state in IOs

**Fig. 1:**
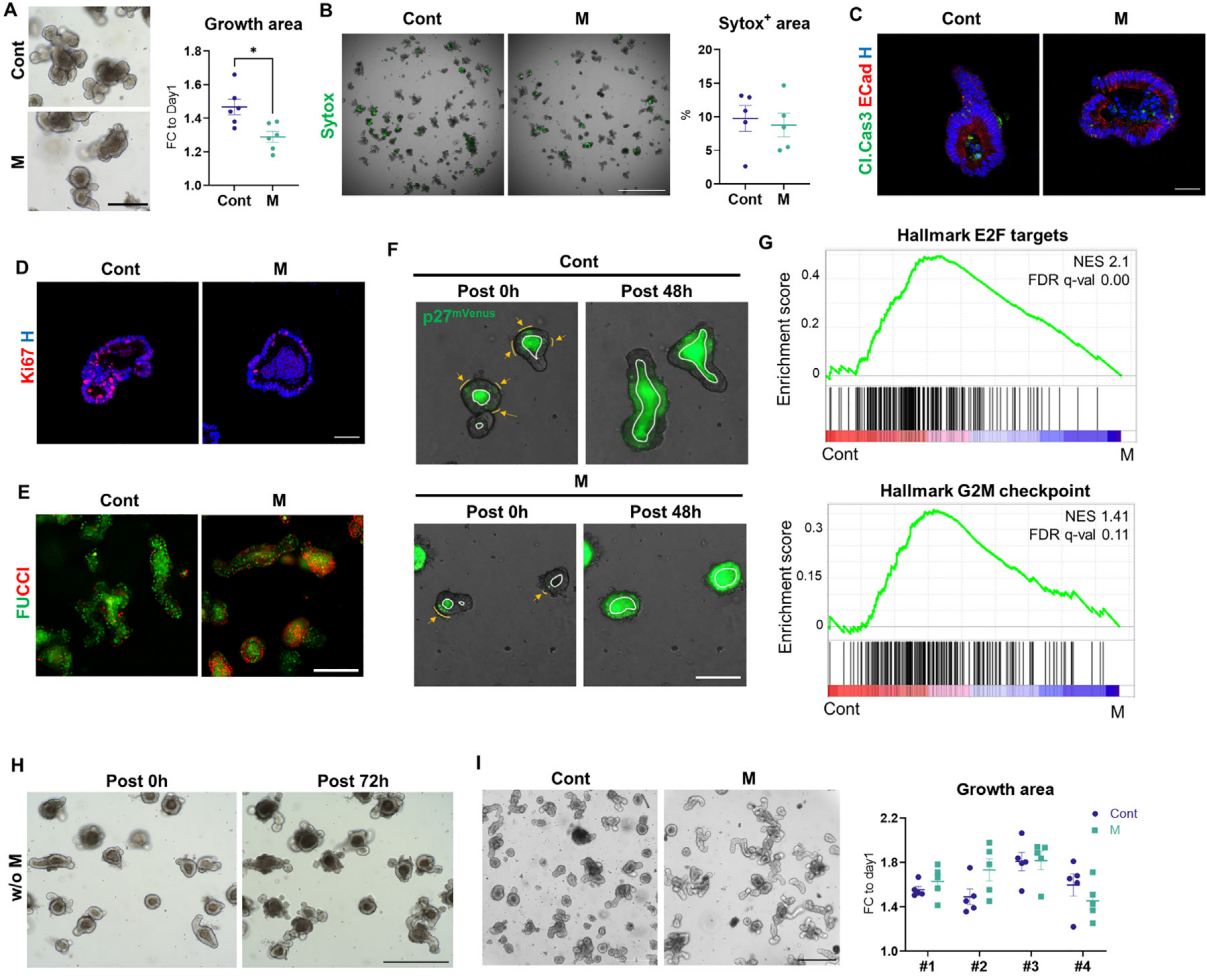
Melatonin induces a quiescent-like state in IOs. (A) Bright-field images of control and melatonin-treated organoids, together with quantitative analysis of organoid growth ratio based on fold change (FC) in the total area occupied by IOs from Day 0 to 4; (B) Organoid viability determined by Sytox staining; (C–D) Immunocytochemistry for cleaved caspase-3 (Cl. Cas3) and Ki67 in IOs; (E) Images of FUCCI reporter organoids showing an increased proportion of G0/G1 (red) cells following MT treatment; (F) Time-lapse fluorescence images depicting the distribution of p27-expressing cells in IOs. p27-expressing cells are indicated by yellow arrows; (G) Transcriptome profiling (microarray) and GSEA for cell cycle gene sets in mSIOs; (H) Bright-field images of organoids 72 h after melatonin withdrawal, indicating re-budding; (I) Quantitative comparison of growth area in subcultured IOs on Day 4. At least two independent organoid lines were used for all experiments. The number of biological replicates corresponds to the number of data points shown in each graph. Scale bars: 500 µm (A), 1000 µm (B), 50 µm (C, D), 200 µm (E, F, H), and 300 µm (I). Data are presented as mean ± SEM and were analyzed using an unpaired *t*-test. *P < 0.05.

First, we investigated the potential impact of melatonin on the fate regulation of intestinal epithelial cells using IO system. Upon melatonin treatment, the typical crypt-villi budding structure diminished in mouse-derived small intestinal organoids (mSIOs), accompanied by a reduction in overall growth area (Fig. 1A), consistent with our previous report [13]. To determine whether this phenomenon resulted from melatonin-induced cytotoxicity, we evaluated cell death in mSIOs. The proportion of nonviable cells labeled with SYTOX staining (Fig. 1B) and the frequency of cleaved caspase-3^+^ apoptotic cells (Fig. 1C) were not affected by melatonin treatment. When cell death was further analyzed by Annexin V/PI staining using FC, no significant difference was observed in the proportion of early apoptotic cells (Annexin V^+^/PI^−^) or late apoptotic- or necrotic cells (Annexin V^+or −^/PI^+^) between groups (Fig. S1A), confirming that melatonin did not compromise organoid viability. Meanwhile, Ki67 immunostaining revealed a reduction in proliferating cells in melatonin-exposed IOs compared with controls (Fig. 1D). Similarly, melatonin treatment increased the proportion of cells in the G0/G1 phase, identified by the red-fluorescent Cdt1 reporter, in IOs expressing the FUCCI system (Fig. 1E). To determine whether melatonin induces a quiescent state in IOs, G0 cells were labeled with a mutant p27 (lacking cyclin-dependent kinase inhibitory activity)-mVenus fusion protein as previously described [24]. Cells exhibiting high mVenus intensity lacked EdU incorporation (Fig. S1B), indicating that p27^hi^ cells represent a rarely-dividing, stable quiescent population in IOs. Tracking fluorescence changes in reporter organoids over 48 h revealed that the emergence of p27-expressing cells markedly increased in the melatonin-treated group (Fig. 1F). Consistent with these findings, Hallmark GSEA showed that pathways linked to cell cycle progression were significantly enriched in control samples compared with melatonin-treated IOs (Figs. 1G and S1C). Collectively, these data demonstrate that melatonin induces quiescence in IOs without exerting cytotoxic effects. Indeed, budding of IOs restarted after melatonin withdrawal from the culture system (Fig. 1H), and melatonin-pretreated organoids exhibited growth patterns and proliferative capacity comparable to those of naïve organoids following passaging (Fig. 1I)

### 3.2 Melatonin contributes to the reprogramming of epithelial cells, enhancing their regenerative capacity

**Fig. 2:**
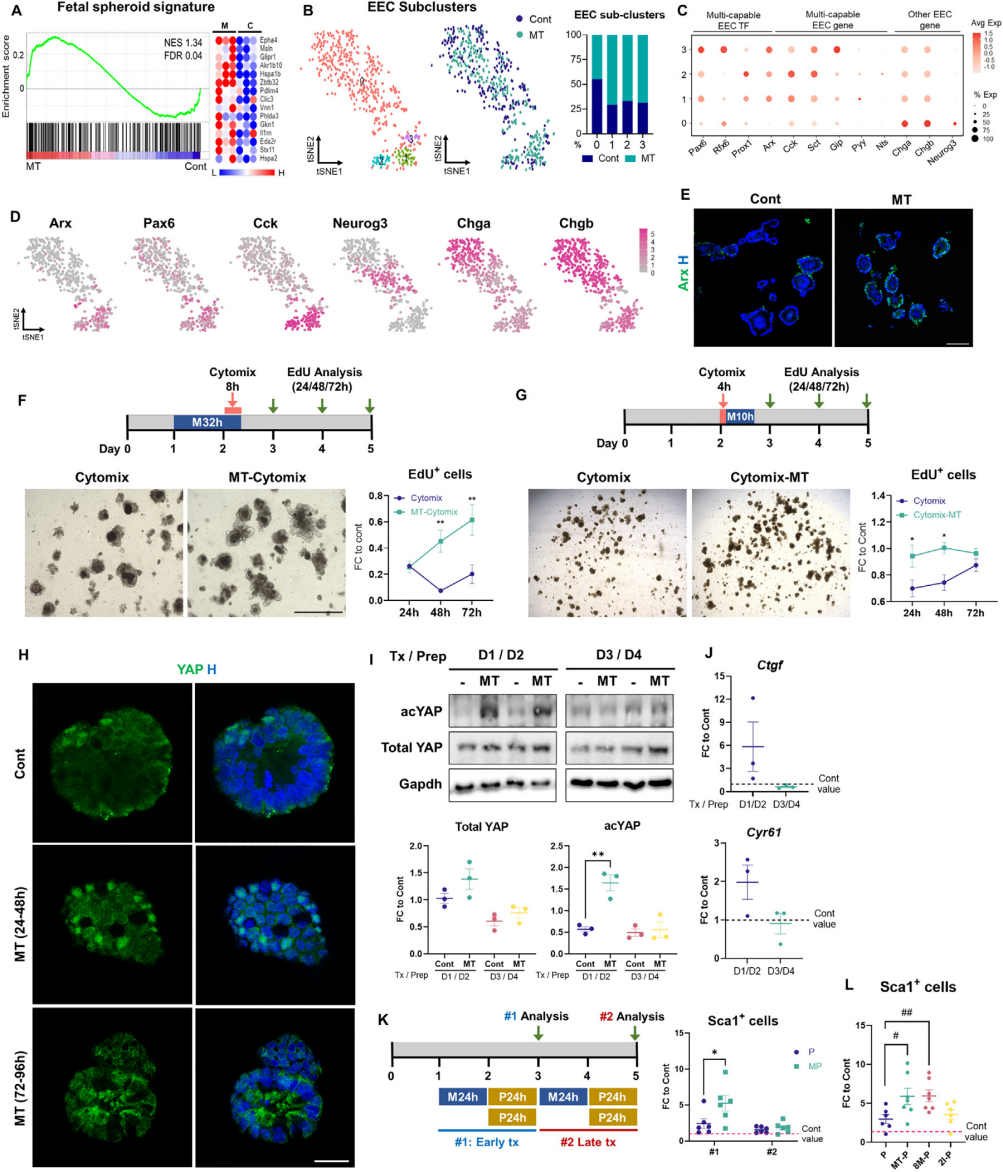
Melatonin enhances regenerative capacity and YAP activation in IOs. (A) GSEA of IOs for a fetal gene signature; (B) t-SNE plots showing cell distribution based on EEC subclusters (left) or treatment groups (right), together with a bar graph indicating the relative proportion of each EEC subpopulation; (C) Dot plot of relative EEC gene expression across subpopulations; (D) t-SNE plots of multicompetent EEC gene expression; (E) Immunofluorescence staining of Arx in IOs. (F, G) Experimental scheme, representative images, and quantification of proliferative capacity in cytomix-exposed IOs following melatonin (F) pre- or (G) post-treatment; (H) Immunofluorescence staining for YAP in IOs demonstrating differential YAP localization after melatonin treatment; (I, J) Time-dependent effects of melatonin on YAP activation: (I) western blots showing total YAP and non-phosphorylated (active) YAP (ac.YAP), with corresponding quantification in IOs, and (J) relative mRNA expression levels of YAP target genes; (K) Experimental design and FC analysis evaluating the induction of Sca1^+^ cells in IOs. (L) FC analysis of the synergy between PGE_2_ and MT1/2 receptor agonists on RSC induction. At least two independent organoid lines were used for all experiments. The number of biological replicates corresponds to the number of data points shown in each graph. Scale bars: 100 µm (E), 200 µm (F), 1000 µm (G), and 20 µm (H). Data are presented as mean ± SEM and were analyzed using an unpaired *t*-test. *P < 0.05, **P < 0.01.

Our previous work indicated that melatonin promotes the EEC population with enhanced stemness [13]. We also observed significant enrichment of a fetal gene signature in melatonin-treated IOs, according to GSEA (Fig. 2A). To further characterize the EECs following melatonin exposure, we revisited our scRNA-seq dataset and performed subclustering of the EEC population (Fig. S2A). Subcluster analysis delineated four distinct populations. Among these, EEC0—the major EEC subpopulation—was detected at similar proportions in both groups, whereas the EEC1–3 subpopulations were increased following melatonin treatment (Figs. 2B and S2B). Considering the established role of differentiated EECs in mediating regeneration in the context of epithelial injury [6,8], we performed differential gene expression analysis between EEC1–3 and EEC0 to investigate differences in stemness among the EEC subclusters. We identified 263 upregulated and 607 downregulated DEGs in EEC1–3 compared with EEC0 (Supplementary Dataset 1). Gene ontology enrichment analysis revealed that transcripts in EEC1–3 were primarily enriched in secretion-associated pathways, whereas those in EEC0 were predominantly associated with cellular metabolic processes, indicating distinct functional characteristics (Supplementary Dataset 1). Of interest, EEC1–3 cells exhibited a gene expression profile characteristic of multipotent secretory precursors, resembling regeneration-driving cell populations [[25], [26], [27]]. In specific, transcripts encoding EEC-secretory factors such as *Cck* and *Sct*, and their transcriptional factors *Prox1, Arx* and *Pax6*—transiently induced in injury-inducible ISCs following radiation [26]—were predominantly enriched in this subpopulation (Fig. 2C and D). Consistent with the increased transcriptional levels of *Arx* and *Pax6* (Fig. S2C), immunofluorescence analysis demonstrated enhanced nuclear Arx expression in melatonin-treated IOs compared with controls (Fig. 2E). Growing evidence suggests that a subset of EECs participates in intestinal epithelial plasticity, facilitating repair through adaptive reprogramming. Indeed, forced induction of quiescence in Lgr5^+^ CBCs by blocking the EGFR pathway biases differentiation toward the EEC lineage [28]. Moreover, the cyclin-dependent kinase inhibitor p57 marks a subset of noncycling EECs that reactivate in response to epithelial damage, thereby driving regeneration in the absence of CBCs [8]. Building on these insights, our results propose a potential contribution of melatonin in rewiring the intestinal epithelium to promote the emergence of a quiescent, intermediate state of facultative stem cells within the EEC population.

These findings prompted us to evaluate the supportive role of melatonin in epithelial regeneration following injury. To this end, IOs were pretreated with melatonin for 24 h prior to cytomix exposure (a mixture of IFN-γ, TNF-α and IL-1β) to mimic inflammatory damage. Following cytomix treatment, IO viability was significantly impaired, as evidenced by disrupted structures and reduced growth; in contrast, melatonin-pretreated IOs exhibited a robust recovery response to injury, displaying enhanced proliferation over time (Fig. 2F). To further evaluate stemness, each group was promptly passaged after cytomix treatment and assessed for growth and proliferation patterns. Notably, the melatonin-pretreated group generated a greater number of organoids with a higher proportion of proliferating cells than untreated counterparts (Fig. 2G). Collectively, these findings indicate that melatonin reprograms intestinal epithelial cells to enhance their regenerative capacity, thereby facilitating more rapid recovery from injury.

### 3.3 Melatonin sustains YAP activation in IOs

RSCs, characterized by a slow-cycling, quiescent state, rely on activation of YAP signaling for their induction and subsequent regenerative responses [10,24]. Building on GSEA-based findings that melatonin enhances YAP activation in IOs (Fig. S2D), we performed a time-course analysis to further elucidate its regulatory mechanism. YAP signaling is dynamically regulated during self-organization and normal development of IOs; in homeostatic mSIOs, it is gradually activated during the first 72 h of culture and subsequently declines [29,30]. Immunofluorescence staining showed that melatonin treatment on Day 1 promoted nuclear localization of YAP compared with the control (Fig. 2H). In contrast, when melatonin was administered on culture Day 3 (a stage known as the YAP-OFF phase), nuclear YAP expression was barely detectable. Consistently, only IOs treated with melatonin on Day 1 exhibited a concurrent increase in both non-phosphorylated (active) YAP and total YAP levels (Fig. 2I), which in turn led to the upregulation of YAP target gene transcription (Fig. 2J).

Given that melatonin potentiates PGE_2_-mediated RSC formation in IOs [13], next we investigated whether the timing-dependent effect of melatonin on YAP activation influences the overall enhancement of RSC generation upon PGE_2_ stimulation. Melatonin pretreatment was administered at early (Day 1) or late (Day 3) time points prior to PGE_2_ stimulation, after which the proportion of Sca1^+^ RSCs (encoded by *Ly6a*) was evaluated by FC. As expected, PGE_2_ alone efficiently induced RSCs under both conditions, although early treatment yielded a higher induction capacity than late treatment (Fig. 2K). Interestingly, synergistic enhancement of PGE_2_-induced RSC formation was observed exclusively in IOs pretreated with melatonin on Day 1 (Fig. 2K). Previous studies have shown that YAP mediates the regenerative effects of melatonin in several organs, including the heart, bone, and intervertebral discs [[31], [32], [33]]. Consistently, our findings support a role for melatonin in regulating YAP dynamics within the intestinal epithelium. Notably, these effects were observed exclusively when melatonin was administered during the early stage of culture (approximately within the first 72 h), a period during which YAP activation is spontaneously induced, [29] suggesting that melatonin does not directly initiate YAP activation but rather modulates its nuclear retention and/or degradation.

Melatonin signaling is primarily mediated through its membrane-bound G protein-coupled receptors (GPCRs), MT1 and MT2 [34]. To delineate the specific melatonin receptor subtype responsible for YAP activation, we utilized 2I and 8M as MT1- and MT2-selective agonists, respectively. First, we treated 2I or 8M to IOs and compared their YAP activation levels to those of control and melatonin-treated groups. Interestingly, 8M recapitulated the action of melatonin on YAP activation (Fig. S3). To further determine whether specific MT2 activation is sufficient to potentiate PGE2-mediated RSC induction *in vitro*, we pretreated organoids with melatonin, 2I or 8M prior to PGE_2_ stimulation and evaluated the emergence of Sca1-expressing cells. Only 8M pretreatment exhibited a synergistic effect on PGE_2_-mediated RSC induction comparable to that of melatonin, while no significant impact was observed with 2I (Fig. 2L). Collectively, these data suggest that MT2 activation is the primary driver of melatonin-mediated YAP activation and its synergistic role in PGE_2_-induced RSC induction. Given that melatonin is a highly pleiotropic molecule, acting not only through membrane-bound GPCRs but also via nuclear receptors and direct transcriptional modulation [34], it remains to be determined whether MT2 serves as the exclusive driver of this process or if complementary intracellular pathways provide functional redundancy. Furthermore, delineating the upstream regulators—particularly the Hippo-LATS kinase cascade—will be essential to fully elucidate how melatonin coordinates YAP activity.

### 3.4 Generation and characterization of melatonin-incorporated 3D-MSCs (Mel-HS)

**Fig. 3:**
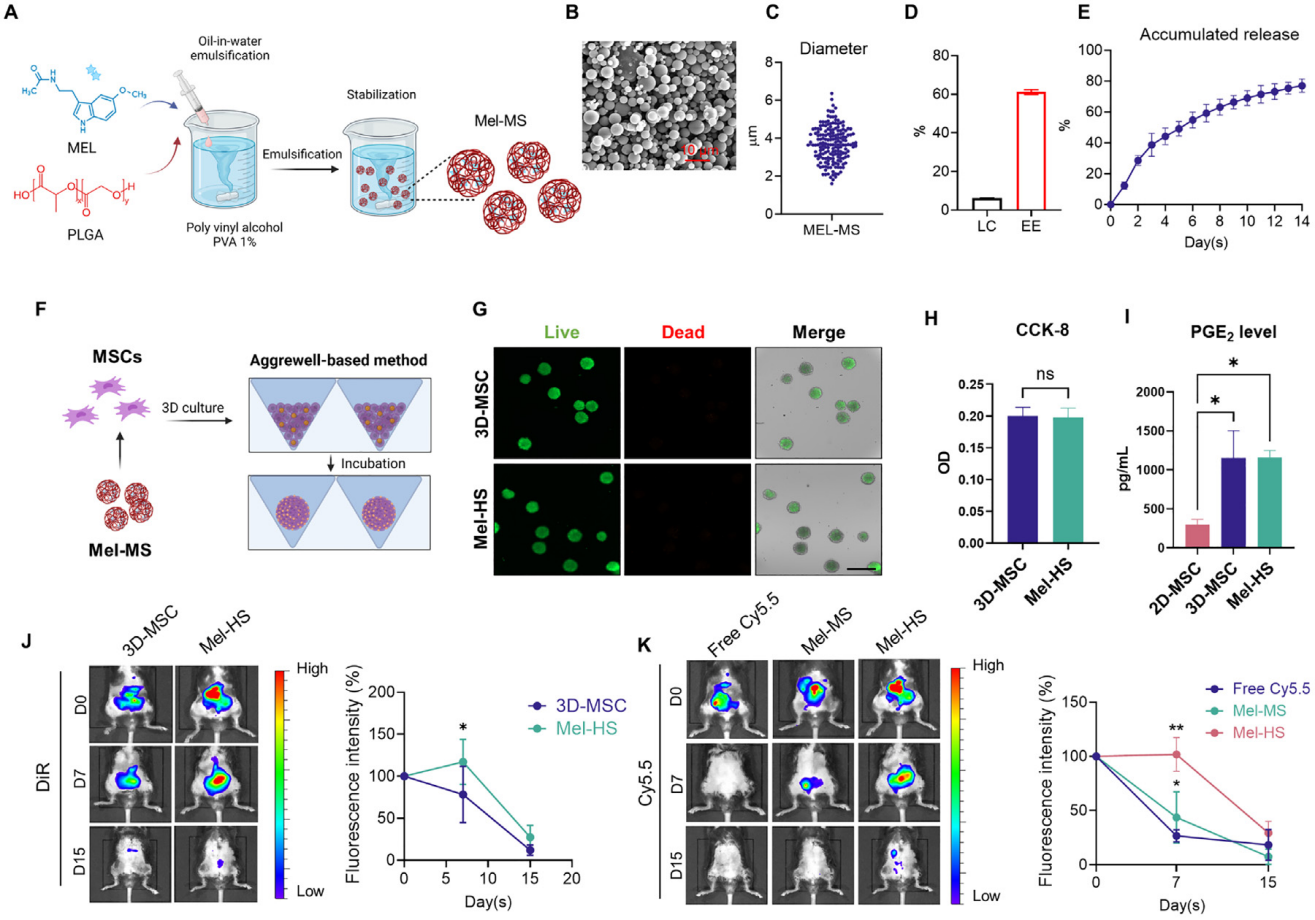
*In vitro* characterization and *in vivo* retention of 3D-MSCs and Mel-HS. (A) Schematic diagram illustrating the preparation procedure of Mel-MS and (B) a representative SEM image; (C–E) Characterization of Mel-MS, including (C) size distribution, (D) LC and EE, (E) and *in vitro* release profile of melatonin from Mel-MS in PBS (pH 7.4; *n* = 3); (F) Schematic diagram depicting formation of Mel-HS; (G) LIVE/DEAD staining of 3D-MSCs and Mel-HS; (H) Cell viability comparison between 3D-MSC and Mel-HS groups; (I) Quantification of PGE_2_ secretion; (J, K) Fluorescence images and quantitative analysis of (J) DiR-labeled MSC signals and (K) MS-derived Cy5.5 signals 2 weeks after intraperitoneal injection. Scale bar: 500 µm. Data are presented as mean ± SEM and were analyzed using an unpaired *t*-test. *P < 0.05, **P < 0.01.

We previously demonstrated that MSCs ameliorate DSS-induced colitis by inducing Claudin4^+^ or Sca1^+^ cells in the intestinal epithelium, a process mediated by MSC-derived PGE_2_ [16]. Inspired by the present organoid-based findings, we designed a complex consisting of MSCs (as a potent source of PGE_2_) and melatonin and evaluated its impact on RSC induction in IOs. To this end, melatonin was first encapsulated in PLGA microspheres (Mel-MS) using an oil-in-water emulsification method (Fig. 3A). SEM exhibited a regular spherical shape and smooth surface, with a size range of 2 to 5 µm (average 3.7 ± 0.9 µm) (Figs. 3B-C and S4A). The mean LC and EE of Mel-MS, as determined by HPLC, were 6.09% ± 1.22% and 60.9% ± 5.7%, respectively (Fig. 3D). Notably, Mel-MS exhibited a sustained release pattern of melatonin for 2 weeks, with a low initial burst release during the first 2 days (Fig. 3E). To better mimic the inflammatory colonic microenvironment, we also evaluated the release kinetics in an acidic medium (pH 5.4), which indicated an accelerated degradation and release profile, reaching approximately 75% release over 8 days (Fig. S4B). Crucially, the Mel-MS platform maintained a sustained-release pattern throughout this period, demonstrating its durability and controlled-release capability under physiologically challenging conditions. Thereafter, the melatonin release profile demonstrated a relatively slow and continuous release, with approximately 80% of the total loaded melatonin released into the medium over 14 days. In the next step, we incorporated Mel-MS during the MSC spheroid (3D) formation step to create Mel-HS (Fig. 3F). The diameter distribution of Mel-HS was slightly bigger (average approximately 162.09 µm) than that of 3D-MSCs (145.42 µm) (Fig. S4C). Live/dead staining and the CCK-8 proliferation assay showed no significant differences between the two groups (Fig. 3G and H). We further demonstrated that PGE_2_ secretion by Mel-HS was comparable to that of 3D-MSCs, and both were greater than that observed in conventional 2D-cultured MSCs (Fig. 3I). These findings confirm that incorporation of Mel-MS did not affect MSC viability or paracrine capacity, at least with respect to PGE_2_ secretion. To investigate the retention time and distribution pattern of 3D-MSCs and Mel-HS after transplantation, MSCs and Mel-MS were prelabeled with DiR and Cy5.5, respectively, prior to fabrication of 3D-MSCs and Mel-HS. Labeled 3D-MSCs, Mel-MS, Mel-HS and free Cy5.5 were then injected intraperitoneally into mice and fluorescent signals were monitored over time. The DiR signal derived from MSCs remained intact even 1 week after injection in Mel-HS-treated mice, whereas it declined by approximately 70% in 3D-treated mice (Fig. 3J). Consistent with this observation, cell aggregates attached to the intestine or colon were observed exclusively in the Mel-HS-treated mice group on Day 15 (Fig. S4D), suggesting that sustained melatonin release from Mel-MS enhances *in vivo* MSC survival, at least in part due to its cytoprotective effects. Intriguingly, Cy5.5 signals derived from Mel-HS were well-preserved until Day 7, whereas signals from free Cy5.5 and Mel-MS alone rapidly diminished, decreasing to less than half over the same period (Fig. 3K). To further validate systemic safety of intraperitoneal Mel-HS injection, we assessed serum biochemical markers (AST, ALT and BUN) in healthy mice. Our results demonstrated that these parameters remained within normal physiological ranges and showed no significant differences compared to the control group, indicating that the local delivery of Mel-HS does not induce systemic liver or kidney toxicity (Fig. S4E). These data collectively indicate that Mel-HS represents an excellent platform with superior *in vivo* retention, attributable to the complementary interaction between MSCs and melatonin.

### 3.5 Mel-HSs enhance the induction of sca1^+^ RSCs and aid recovery in damaged IOs

**Fig. 4:**
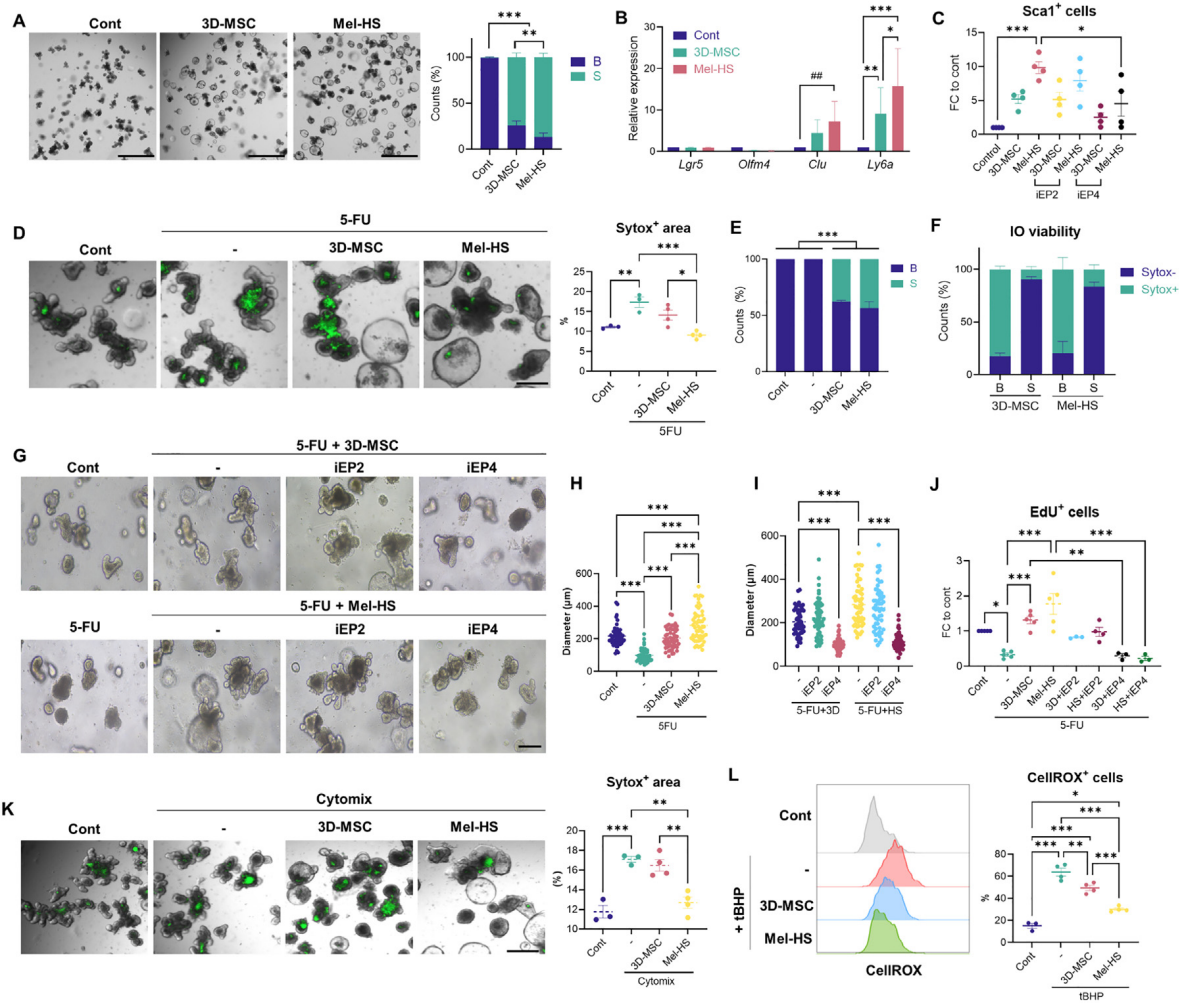
Effect of Mel-HS on RSC generation and epithelial protection *in vitro*. (A) Bright-field images of IOs co-cultured with CM derived from 3D-MSCs and Mel-HS, with quantitative classification of organoid morphology into typical budding organoids and spheroids; (B) mRNA expression levels of CBC markers (*Lgr5, Olfm4*) and RSC markers (*Clu, Ly6a*) in IOs; (C) Dot plot showing quantification of Sca1^+^ cells in IOs by FC; (D–F) Sytox staining–based analysis of organoid viability following 5-FU treatment: (D) Bright-field images with corresponding quantification of Sytox-positive area, (E) morphological assessment with (F) quantification of the proportion of Sytox^+^ IOs; (G–J) Effects of CM and EP antagonists on growth of 5-FU–exposed organoids after subculture: (G) Bright-field images of organoid morphology 72 h after passaging, (H, I) organoid growth, and (J) proliferation assessed by EdU incorporation; (K) Organoid viability following proinflammatory stimulation; (L) FC analysis of oxidative stress levels in IOs. The number of biological replicates corresponds to the number of data points shown in each graph. Scale bars: 1000 µm (A) and 200 µm (D, G, K). Data are presented as mean ± SEM and were analyzed using two-way ANOVA with Tukey’s multiple-comparisons test. *P < 0.05, **P < 0.01, ***P < 0.001. For (B), ^##^*P* < 0.01, statistical significance was determined using an unpaired *t*-test.

Next, we investigated the effect of Mel-HS on RSC induction in IOs. Morphological assessment showed that the emergence of cystic organoids, a typical response to PGE_2_ treatment [11,16], was facilitated not only by direct co-culture with 3D-MSCs or Mel-HS, but also by treatment with their conditioned media (CM) (Figs. 4A and S5A). Notably, organoids co-cultured with Mel-HS or its CM exhibited the highest expression levels of the RSC marker genes *Ly6a* and *Clu* compared with all other groups (Figs. 4B and S5B). FC analysis further confirmed that Mel-HS most effectively induced Sca1^+^ cells (Figs. 4C and S5C). In addition, co-treatment with an EP4 inhibitor, but not an EP2 inhibitor, successfully attenuated the ability of CM to induce RSCs in IOs (Fig. 4C), highlighting the indispensable role of MSC-derived PGE_2_ in this process. These observations indicate that Mel-HS recapitulates the synergistic effect of the naïve melatonin-PGE_2_ combination in promoting intestinal RSC induction.

We then evaluated the protective effects of this biohybrid against various epithelial insults (Fig. S5D–5G). To deplete homeostatic ISCs, organoids were treated with 5-FU, which targets actively proliferating cells by inhibiting DNA/RNA synthesis. After 24 h exposure, organoids displayed a marked increase in Sytox-positive cells, indicative of nonviable cells (Fig. 4D). Of note, co-treatment with CM from Mel-HS (HS-CM) substantially reduced cell death under 5-FU challenge (Fig. 4D). Morphological analysis showed that CMs derived from both 3D-MSCs and Mel-HS induced spheroid transformation in 5-FU-treated IOs (Fig. 4E). Interestingly, spheroid-type organoids induced by CM treatment demonstrated greater resistance to ISC depletion than budding organoids, highlighting the enhanced resilience of the RSC-enriched organoids (Fig. 4F). The detrimental impact of 5-FU on the stem cell population became more evident after subculture, as organoids exhibited impaired budding and collapsed epithelial structures (Fig. 4G and H). In contrast, HS-CM treatment elicited a robust recovery response, with the average diameter increasing approximately three folds and exceeding the growth observed in untreated controls (Fig. 4G and H). This regenerative advantage of HS-CM was markedly reduced by an EP4 antagonist, whereas EP2 inhibition had no significant effect (Fig. 4G and I). Consistently, the HS-CM-induced increase in proliferating (EdU^+^) cells was significantly attenuated by EP4 blockade (Fig. 4J). In an *in vitro* IBD-mimicking model induced by cytomix treatment, the marked increase in the Sytox^+^ area was exclusively mitigated by HS-CM, indicating that Mel-HS confers protection against nonspecific, inflammatory damage (Fig. 4K). Finally, given the well-established antioxidative role of melatonin, we examined whether Mel-HS retains this effect following formulation. CellROX sensor-based quantification of endogenous ROS levels indicated that both Mel-HS and its CM were more effective in reducing oxidative stress in tBHP-treated organoids than other treatments (Fig. 4L). We further confirmed that CM-mediated protection was recapitulated by direct co-culture of Mel-HS with damaged IOs (Fig. S5H and 5I). Taken together, these data demonstrate that Mel-HS exhibits a superior capacity not only for RSC induction but also for protection against inflammation- and oxidative stress-mediated injury in IOs compared with naive MSCs.

### 3.6 Mel-HS provides a potent protective effect against DSS-induced colitis

**Fig. 5:**
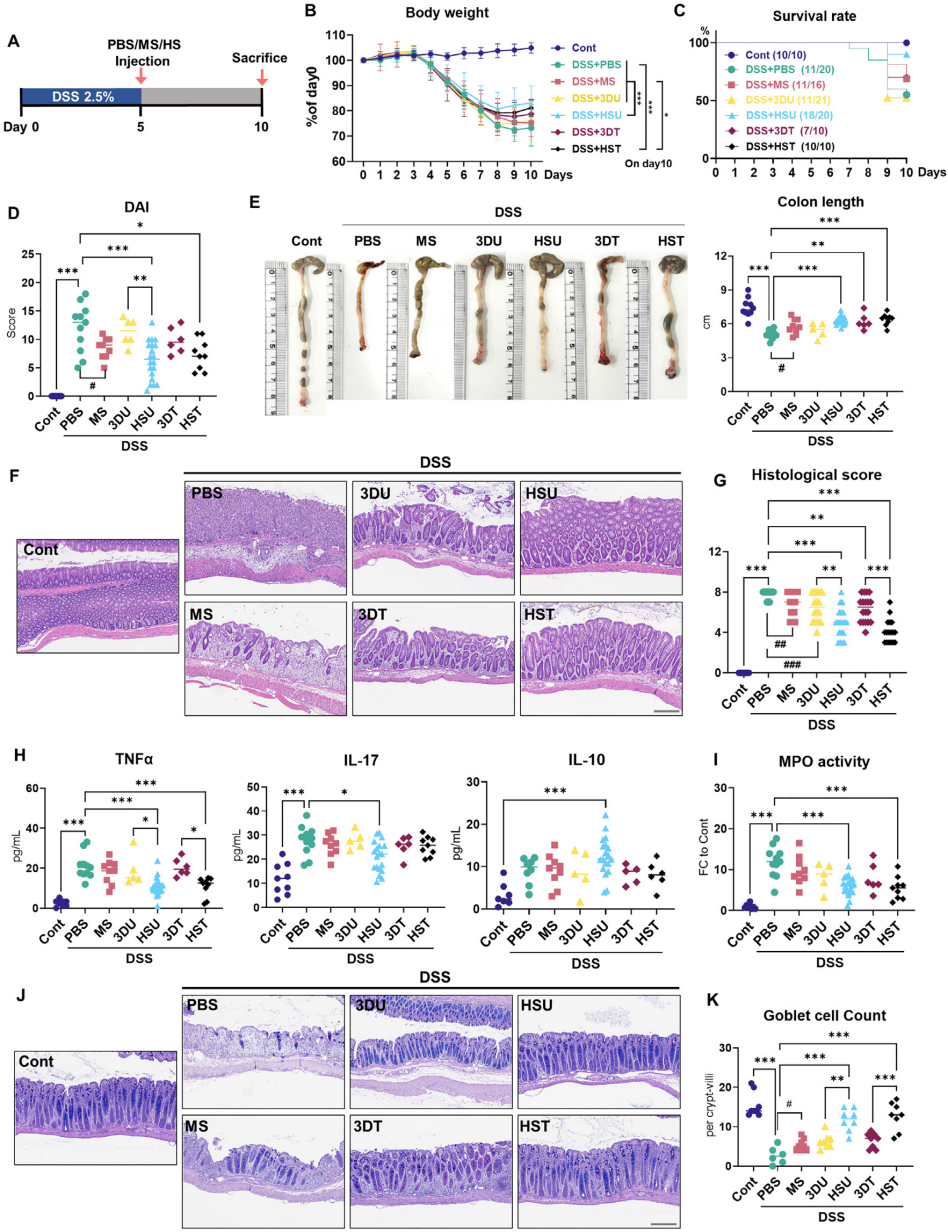
Administration of 3D-MSCs and Mel-HS confers protection against DSS-induced colitis. (A) Experimental timeline; (B) Monitoring of body weight changes and (C) survival rates over 10 d. In (C), the number of surviving mice relative to the total is indicated in parentheses next to each group label; (D) Dot plot showing disease activity index (DAI) scores calculated on Day 10; (E) Comparison of colon lengths among groups; (F) H&E-stained colon sections and (G) corresponding histological scores; (H) Quantification of TNF-α, IL-17 and IL-10 levels in colon tissues by ELISA; (I) Assessment of MPO activity in colon tissues; (J) AB-PAS-stained colon sections from control and DSS-treated mice and (K) quantification of AB-PAS-positive goblet cells per crypt–villus axis. The number of biological replicates corresponds to the number of data points shown in each graph. Scale bar: 500 µm. Data are presented as mean ± SEM and were analyzed using two-way ANOVA with Tukey’s multiple-comparisons test. *P < 0.05, **P < 0.01, ***P < 0.001. For (K), ^#^*P* < 0.05; statistical significance was determined using an unpaired *t*-test.

To determine whether the *in vitro* advantages of Mel-HS over MSCs in protecting the intestinal epithelium are reproducible *in vivo*, 3D-MSCs and Mel-HS were administered in a DSS-induced colitis model and therapeutic outcomes were assessed. In the first experimental setting, injections were performed 1 day after initiation of DSS treatment (Fig. S6A) to evaluate preventive impact. Under these conditions, both 3D-MSCs and Mel-HS led to similarly improved body weight and DAI scores in DSS-treated mice (Fig. S6B and S6C). Next, injections were performed on Day 5, when substantial intestinal damage had developed by DSS exposure, to assess their reparative capacity (Fig. 5A). In this experiment, MSCs derived from either UCB or tonsils (T) were utilized to demonstrate the broad applicability and source-independent efficacy of our therapeutic strategy. Following the verification of their typical CD marker profiles (Fig. S7A) and PGE_2_ secretory levels (Fig. S7B), these cells were incorporated into 3D spheroids (3DU and 3DT) and Mel-HS constructs (HSU and HST). Following DSS administration, the PBS-injected group exhibited continuous weight loss, reaching an approximate 26.7% reduction by Day 10 (Fig. 5B). Notably, Mel-HS–treated mice showed significantly attenuated weight loss compared with all other groups, including the 3D-MSC–treated groups, with HSU demonstrating the most pronounced protection (16.9% reduction) (Fig. 5B). Mel-HS treatment resulted in the highest survival rates among all groups (90% for HSU and 100% for HST) (Fig. 5C). The superior efficacy of Mel-HS in mitigating DSS-induced toxicity compared with 3D-MSCs was further confirmed by DAI scores and colon length measurements (Fig. 5D and E). Histological evaluation of DSS-exposed colons on Day 10 using H&E staining revealed severe epithelial disruption and inflammatory cell infiltration in the PBS-treated group, whereas both 3D-MSC and Mel-HS treatment led to a prominent restoration of epithelium (Fig. 5F and G).

To further characterize the differences in immune responses among groups, representative inflammatory indicators were measured in colon tissue. Elevated levels of the proinflammatory cytokine TNF-α in the PBS group were markedly reduced following Mel-HS treatment, with a greater reduction than that observed in the 3D-MSC group (Fig. 5H). Specifically, administration of HSU to DSS-treated mice normalized the aberrant increase in IL-17 levels while inducing the highest production of the immunomodulatory cytokine IL-10 in the colon (Fig. 5H). We also assessed leukocyte infiltration in colon tissues using an MPO activity assay and found that Mel-HS treatment effectively reduced the excessive MPO activity triggered by DSS administration (Fig. 5I). These findings suggest that Mel-HS possesses superior immunomodulatory capacity compared with Mel-MS and MSCs, effectively attenuating inflammation even after the onset of tissue damage. In addition, we performed AB-PAS staining of colon tissues and quantified mucin-producing goblet cells. While DSS administration significantly reduced the goblet cell population, treatment with 3D-MSCs and Mel-HS increased these cells by more than twofold and fourfold, respectively (Figs. 5J-K and S8). It is worth noting that Mel-MS alone exhibited ameliorative impacts on certain parameters, which were further enhanced when administered in combination with MSCs.

### 3.7 Mel-HS enhances regenerative capacity and restoration of epithelial architecture following DSS treatment

**Fig. 6:**
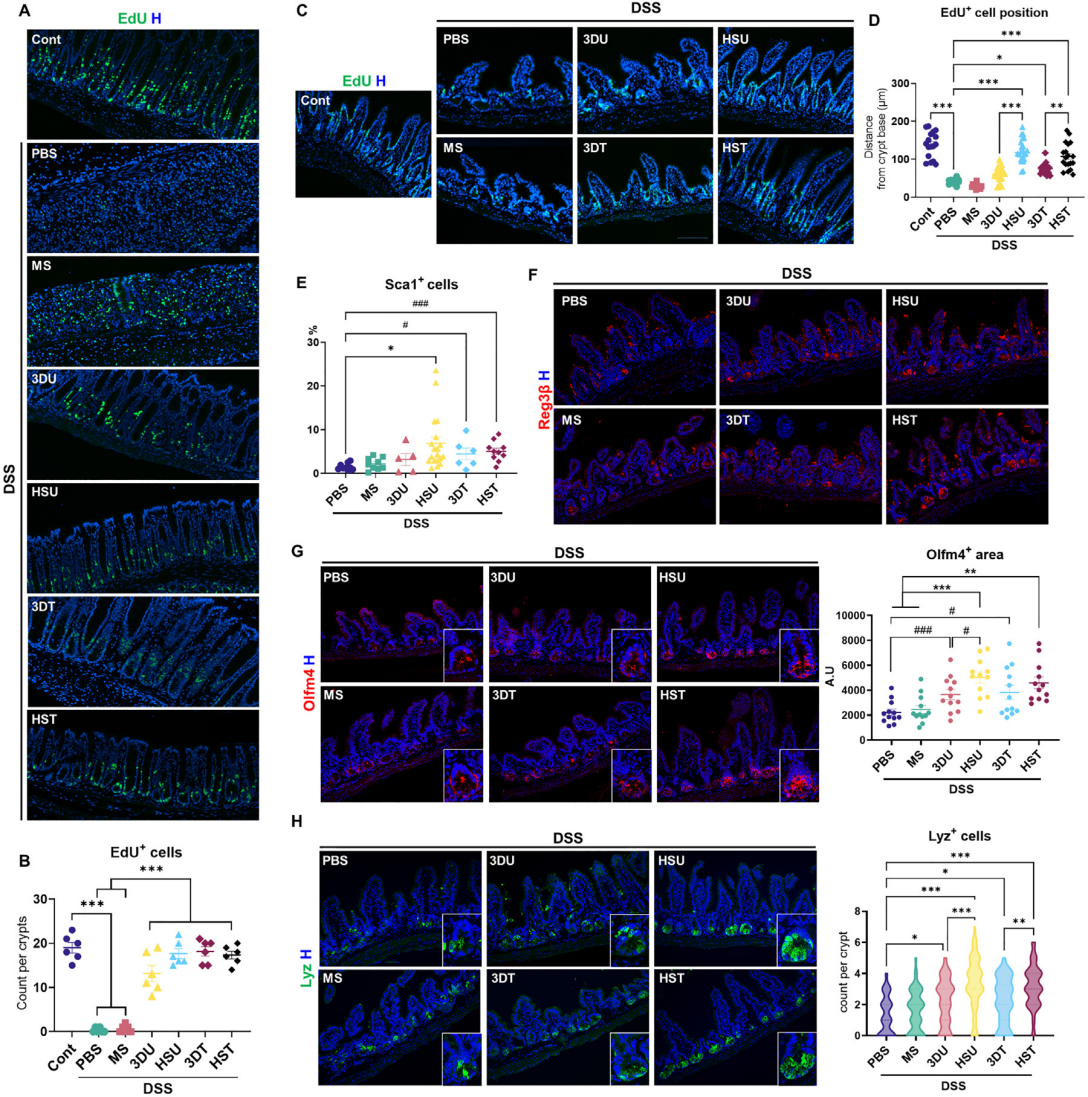
Administration of 3D-MSCs and Mel-HS accelerates the restoration of epithelial architecture in DSS-induced colitis mice. (A) Representative images of EdU-labeled colon sections and (B) quantification of crypts containing EdU-positive cells; (C) Representative images of EdU-labeled ileal sections; (D) Assessment of epithelial cell migration capacity; (E) Quantification of Sca1^+^ cell frequency in crypt-enriched ileal tissues by FC; (F) Immunohistochemical analysis of Reg3β expression in ileal sections; (G-H) Comparison of crypt architecture among groups based on immunofluorescence staining for (G) olfm4 and (H) lysozyme. The number of biological replicates corresponds to the number of data points shown in each graph. Scale bar: 500 µm. Data are presented as mean ± SEM and were analyzed using two-way ANOVA with Tukey’s multiple-comparisons test. *P < 0.05, **P < 0.01, ***P < 0.001. For (E) and (G), ^#^*P* < 0.05 and ### *P* < 0.001; statistical significance was determined using an unpaired *t*-test.

Based on the significant amelioration of epithelial injury observed in 3D-MSC and Mel-HS-treated mice during histological assessment, we hypothesized that epithelial regenerative responses were enhanced in these groups. To evaluate the proliferative capacity of the intestinal epithelium, we labeled actively-dividing cells with EdU for 24 h. On Day 10 after the onset of DSS administration, EdU^+^ cells were sparsely detected within disorganized crypt structures in the colon of the PBS- and Mel-MS-treated groups (Fig. 6A). In contrast, treatment with 3D-MSCs or Mel-HS restored proliferative activity in crypt regions of DSS-affected colons, as evidenced by immunofluorescence and quantification of EdU-positive crypts (Fig. 6A and 6B). In the PBS-treated group, DSS exposure severely disrupted crypt architecture in the colon, rendering quantitative analysis challenging. Therefore, we shifted our focus to the ileum of the small intestine, which, although less affected, still exhibited substantial damage following DSS treatment (Fig. S9A). To estimate the migration capacity of proliferating cells, the distance from the crypt base to the EdU^+^ region was measured. Mel-HS stimulated migration of EdU-labeled cells along the villi to a greater extent than other treatments, including 3D-MSCs (Fig. 6C and 6D). This finding prompted further investigation into the contribution of Mel-HS to acquisition of regenerative features in the injured epithelium. Crypt epithelial cells were enriched from the ileum, and quantitative analysis of Sca1 expression was performed using FC. Administration of 3D-MSCs increased the frequency of Sca1^+^ cells in DSS-exposed crypts by more than two-fold compared with the PBS-treated group; this effect was further enhanced (up to four-fold) when cells were co-administered with Mel-MS (Fig. 6E). Immunohistochemical analysis of Reg3β, a representative marker of regenerating intestine [10,35], revealed increased Reg3β immunoreactivity in both crypt and villus regions of the ileum following treatment with either 3D-MSCs or Mel-HS, compared with PBS or Mel-MS (Fig. 6F). Furthermore, the severe loss of Lyz^+^ Paneth cells and Olfm4^+^ CBC cells observed after DSS exposure was most effectively restored in the Mel-HS-treated ileum (Figs. 6G, 6H and S9B).

Recent studies have shown that forced dedifferentiation via transient activation of reprogramming factors OSKM (Oct4, Sox2, Klf4 and c-Myc) or stimulation of YAP signaling through treatment with epigenetic modulators promotes regenerative responses in the gut epithelium [35,36]. Similarly, our findings support the therapeutic potential of potentiating endogenous RSC activation to treat intestinal injury. It is worth noting that while both 3D-MSCs and Mel-HS provide comparable prophylactic protection, the superiority of Mel-HS becomes markedly pronounced in the therapeutic model. This discrepancy indicates that although the immunomodulatory and antioxidative properties of MSCs and melatonin provide effective protection during early inflammation, the primary advantage of Mel-HS is manifested during the post-injury repair phase, driven by the activation of latent regenerative programs [37], [38]. Given these complementary mechanisms, we anticipate that Mel-HS will not only serve as a potent standalone treatment but also as an effective adjunct to standard-of-care treatments, including 5-ASA and biologics. By integrating mucosal healing with the broad immune suppression provided by these conventional modalities, the Mel-HS platform could overcome the limitations of current IBD management. Moreover, modification of the microsphere formulation with enteric coatings, such as Eudragit [39], can further enhance their delivery efficiency and bioactivity.

While the Mel-HS platform demonstrates enhanced intestinal retention and potent regenerative effects in the DSS-induced colitis model, this study has certain limitations that warrant further investigation. First, while we confirmed systemic safety through serum biochemical markers and observed localized colonic engraftment, a longitudinal quantitative biodistribution and pharmacokinetic analysis remains to be established. Second, although the therapeutic outcomes correlate with the delivery of melatonin and PGE_2_, future studies employing radiolabeling or advanced longitudinal *in vivo* imaging are required to precisely delineate the elimination kinetics of the microspheres and to further characterize the intraluminal concentration gradients. Finally, expanding these safety and pharmacokinetic evaluations to larger animal models or non-human primates will be an essential step toward confirming the long-term safety profile and determining the optimal therapeutic dosage for clinical translation.

## 4 Conclusions

In summary, we demonstrate that melatonin promotes rewiring of the intestinal epithelium toward a more fetal-like, regenerative phenotype that can be reprogrammed into RSCs upon PGE_2_ stimulation, thereby providing insight into a novel role of melatonin in intestinal epithelial regeneration. In a DSS-induced colitis model, combined application of Mel-MS and 3D-MSCs enhances endogenous regenerative responses following epithelial injury, promoting restoration of crypt architecture and proliferative–migratory dynamics along the crypt–villus axis, thereby accelerating epithelial healing. By highlighting the *in vivo* therapeutic efficacy of Mel-HS as a potent inducer of RSCs, our study proposes an innovative treatment strategy that enhances endogenous regenerative potential in IBD and other epithelial defects, broadening the scope of regenerative medicine and cell-based therapy.

## CRediT authorship contribution statement

**Yoojin Seo:** Conceptualization, Investigation, Data curation, Writing – original draft. **Ji-Su Ahn:** Conceptualization, Investigation, Data curation, Writing – original draft. **Nhu-Nam Nguyen:** Investigation, Data curation, Writing – original draft. **Hyeon Seo Lee:** Investigation, Formal analysis, Methodology. **Yunji Lee:** Investigation, Formal analysis, Methodology. **Seong Hui Kim:** Investigation, Formal analysis, Methodology. **Jeong Hyun Yu:** Investigation, Formal analysis, Methodology. **Ji-Won Yang:** Visualization. **Hee-Jeong Park:** Visualization. **Hansong Lee:** Formal analysis. **Tae-Hoon Shin:** Investigation, Formal analysis, Methodology. **Byung-Chul Lee:** Investigation, Formal analysis, Methodology. **Eui-Suk Sung:** Validation. **Jung-Hwan Lee:** Validation. **Won Kyu Kim:** Validation. **Jung-Min Oh:** Methodology. **Dongjun Lee:** Methodology. **Yun Hak Kim:** Formal analysis. **Jee-Heon Jeong:** Conceptualization, Project administration, Supervision, Writing – review & editing. **Hyung-Sik Kim:** Conceptualization, Project administration, Supervision, Writing – review & editing.

## Declaration of competing interest

The authors declare that there is no conflict of interest.

## Appendix Supplementary materials

Image, application 1

Image, application 2
