# Supplementary material for: Melatonin-inspired hybrid spheroids accelerate intestinal repair via YAP-driven fetal reprogramming
Source: Asian J Pharm Sci. 2026 Jun 20;21(4):101176. doi: 10.1016/j.ajps.2026.101176 (PMC13400227; doi:10.1016/j.ajps.2026.101176)
Supplement: Supplementary file 1 [file mmc1.docx]

**Supplementary Materials**

**Melatonin-inspired hybrid spheroids accelerate intestinal repair via YAP-driven fetal reprogramming**

Yoojin Seo^a,#^, Ji-Su Ahn^a,#^, Nhu-Nam Nguyen^b,#^, Hyeon Seo Lee^a,c,d^, Yunji Lee^a,c,d^, Seong Hui Kim^a,c,d^, Jeong Hyun Yu^a,c,d^, Ji-Won Yang^a^, Hee-Jeong Park^a,c,d^, Hansong Lee^e^, Tae-Hoon Shin^f^, Byung-Chul Lee^g^, Eui-Suk Sung^h^, Jung-Hwan Lee^i^, Won Kyu Kim^j,k^, Jung-Min Oh^a^, Dongjun Lee^l^, Yun Hak Kim^m^, Jee-Heon Jeong^b,*^, and Hyung-Sik Kim^a,c,d,*^

**
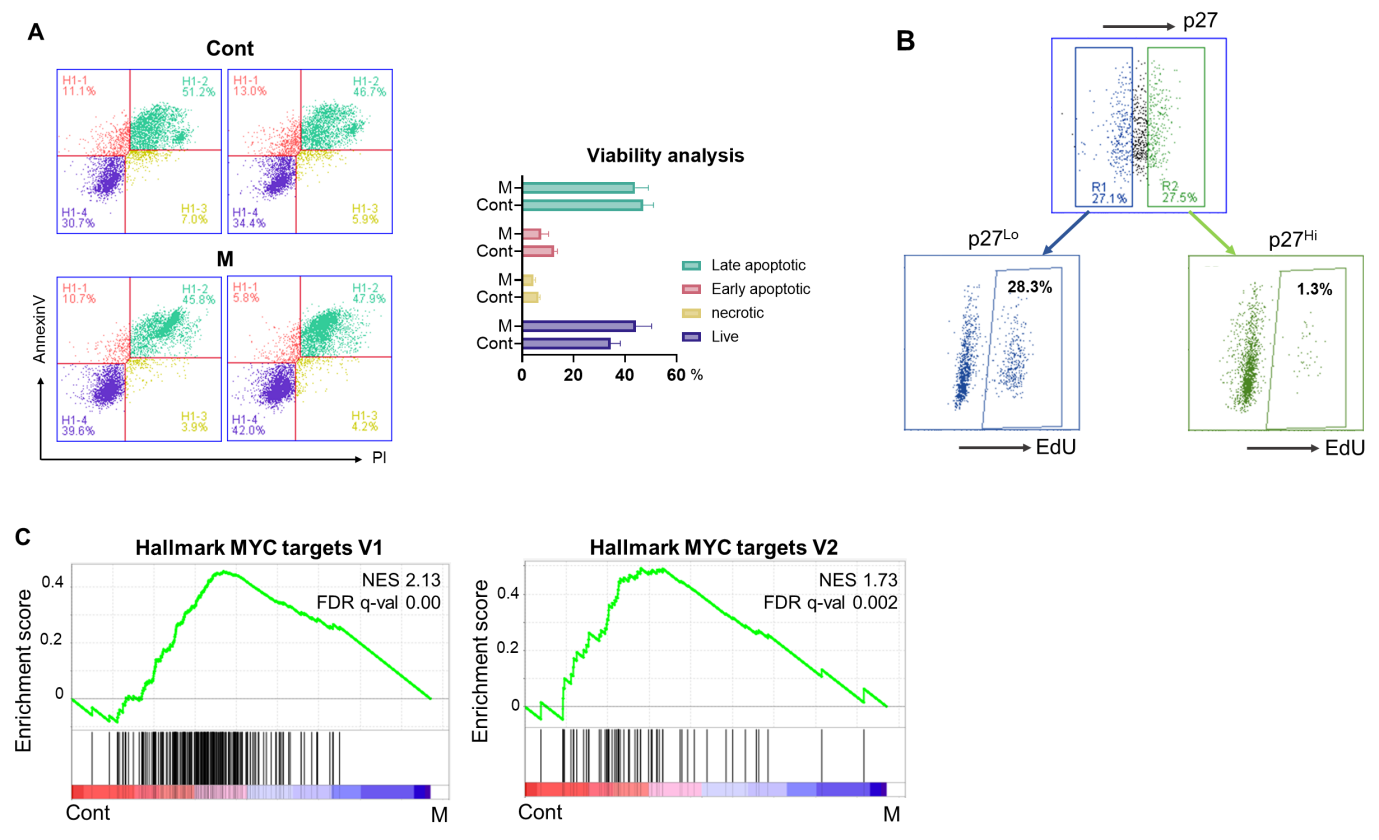
 Figure S1**. **Melatonin treatment affects cell cycle of IOs**. (A) Flow cytometric analysis for AnnexinV/PI stained IOs. (B) Gating and EdU detection images for p27^high^ and p27^low^ cells. (C) GSEA of Contol- and melatonin-treated IOs for cell proliferation-related terms.

**
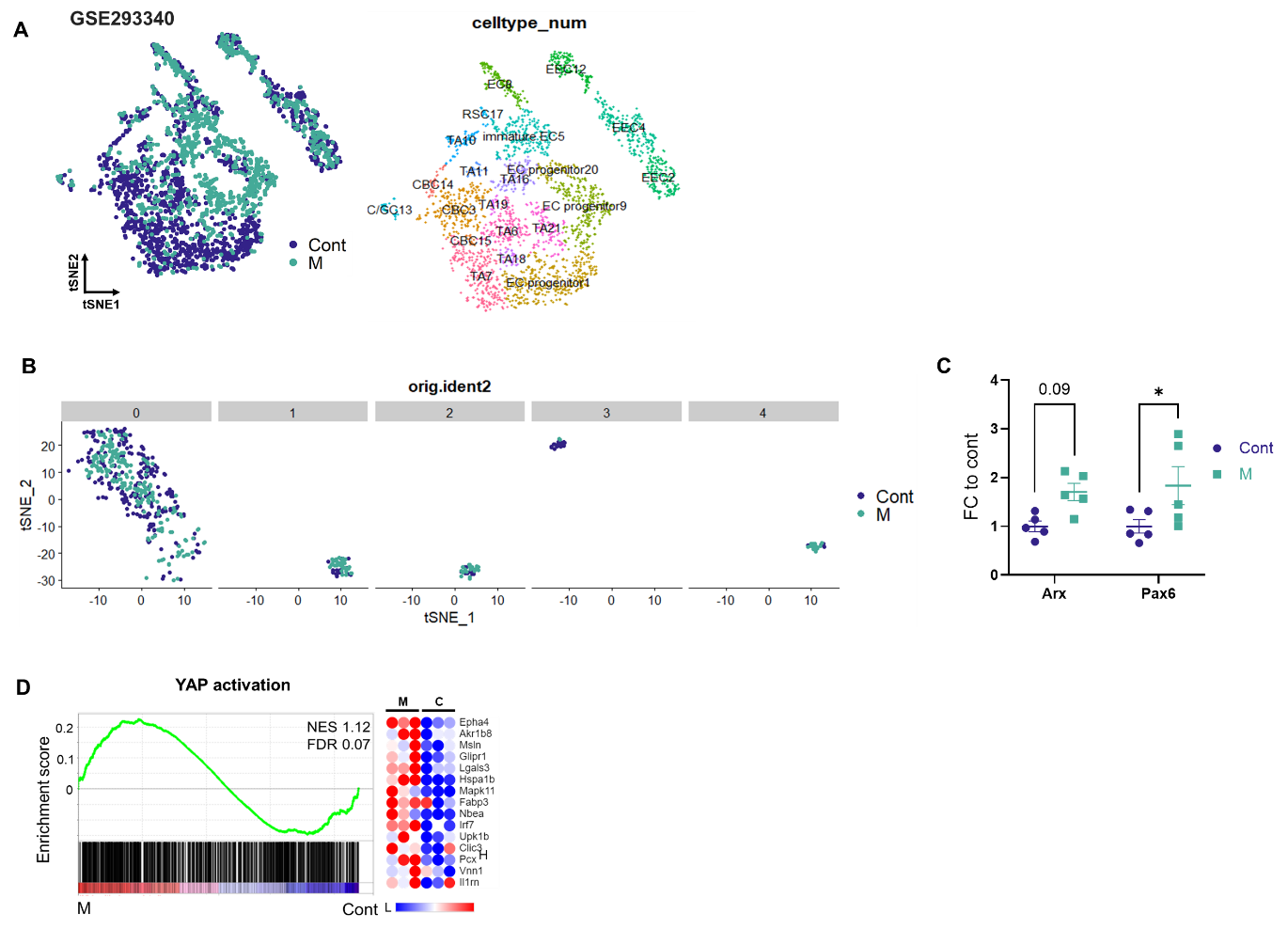
**

**Figure S2**. **Cell proliferation status and regenerative features in melatonin treated IOs.** (A) The t-SNE plot of cont- and melatonin-treated IOs extracted from GSE293340, colored by the group, cell type and cluster number. (B) the t-SNE plot of EEC subclusters. (C) Quantification of mRNA levels of Arx and Pax6 in control- and Melatonin-treated IOs. (D) GSEA of Contol- and melatonin-treated IOs for YAP activation-related genes. Data are shown as the mean ± SEM and compared by unpaired t-test. *P < 0.05.


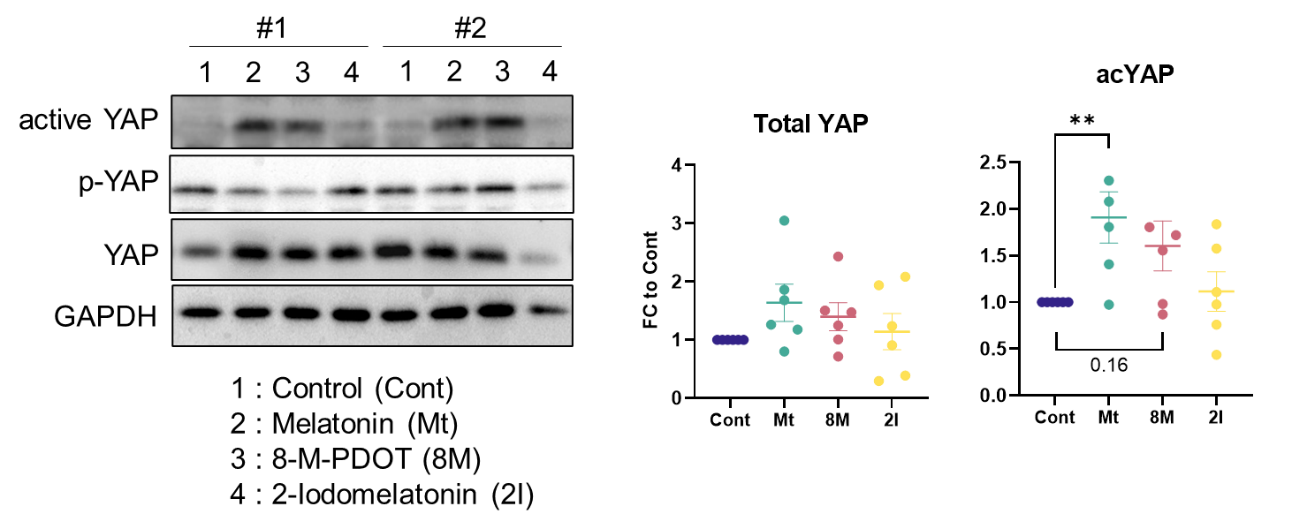


**Figure S3. MT2 signaling is the primary mediator of melatonin-induced YAP activation.** Representative western blotting bands accompanied with corresponding quantification of total- and non-phosphorylated YAP expression. Data are shown as the mean ± SEM and compared by One-way ANOVA with Tukey's multiple comparisons test. **P < 0.01.


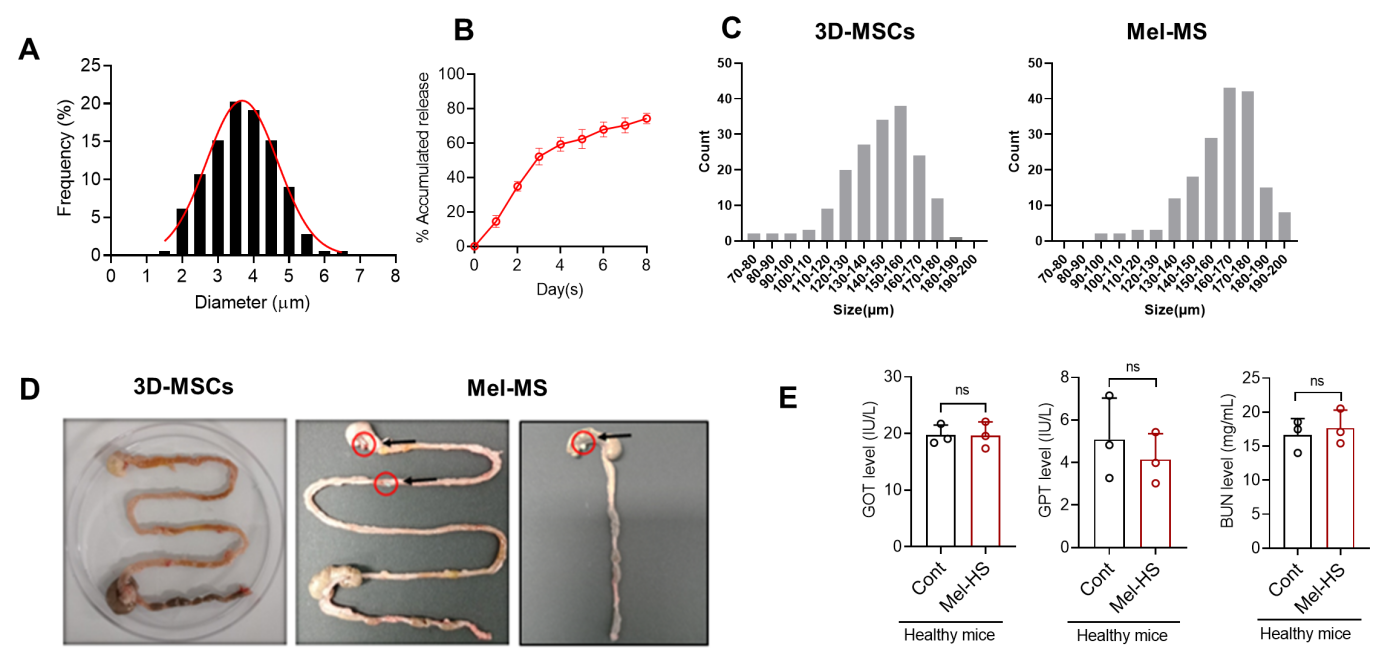


**Figure S4**. **In vitro characterization, in vivo retention assay of Mel-MS and Mel-HS.** (A) The size distribution of Melatonin-loaded microsphere. (B) Release profile of Mel-MS in pH 5.4 (n = 3). (C) The size distribution of 3D-MSC and Mel-HS. (D) Cell aggregates formation after intraperitoneal injection of Mel-MS. (E) Concentration of GOT, GPT, and BUN in the blood serum of sacrificed mice at day 15 post-administration (n = 3).


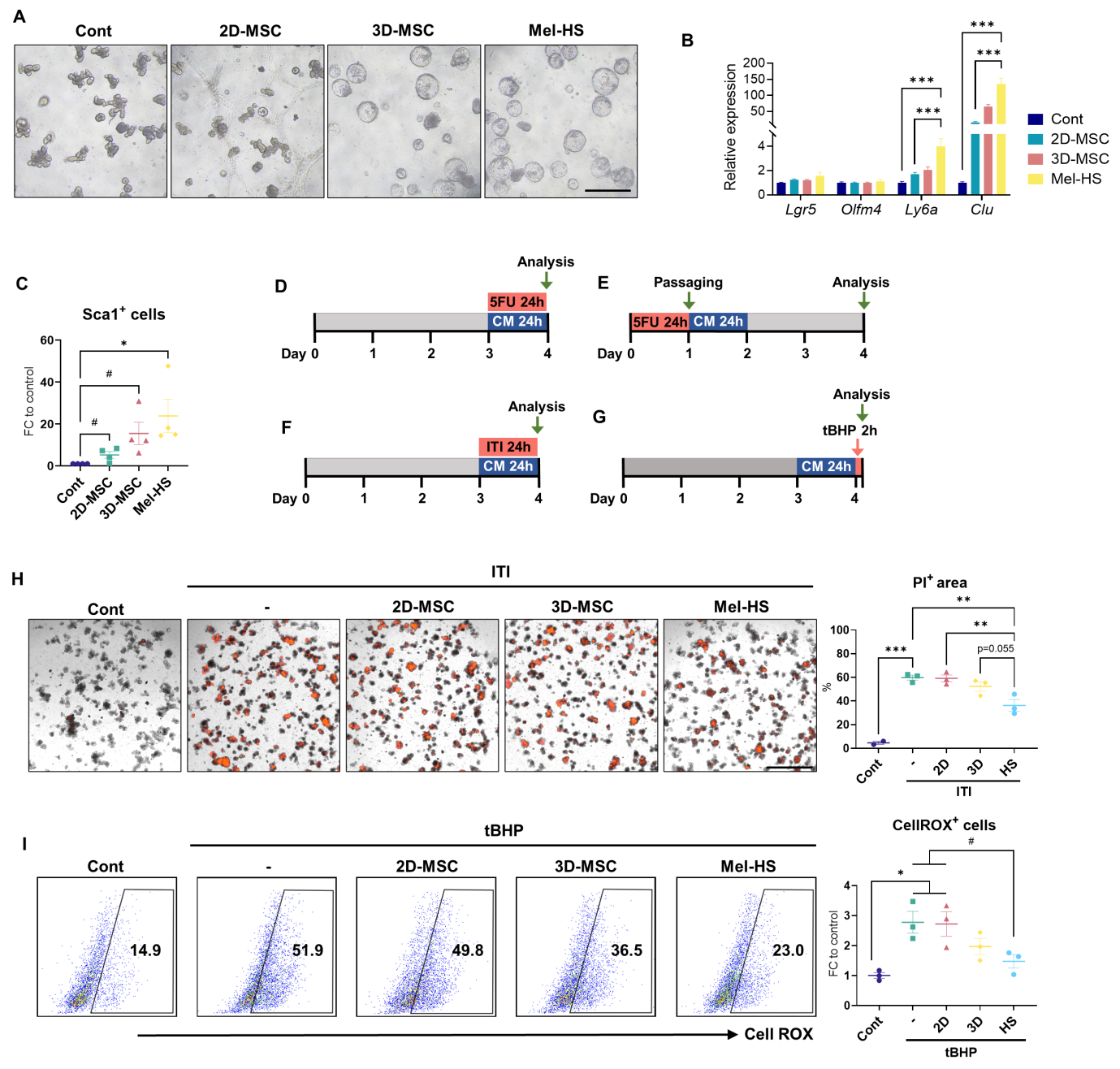


**Figure S5**. **Evaluation of protective effects of 3D-MSC and Mel-HS on injured IOs in a direct co-culture system.** (A) Representative bright-field images of mSIOs co-cultured with 2D-MSC, 3D-MSC, and Mel-HS for 48 hours. (B) Measurement of mRNA expression levels for CBC (Lgr5, Olfm4) and RSC (Clu, Ly6a) markers in mSIOs upon direct co-culture. (C) Quantification of Sca1+ cells using flow cytometry. (D-G). Experimental design for organoid Injury and repair assays related to figure 4. (H) PI staining-based analysis of cell damage in mSIOs cultured with 2D-MSC, 3D-MSC, and Mel-HS following the exposure to a pro-inflammatory combination. (I) CellROX staining-based flow cytometry analysis of oxidative stress in mSIOs cultured with 2D-MSC, 3D-MSC, and Mel-HS following the treatment with tBHP. Scale bar = 200 µm. Data are shown as the mean ± SEM and compared by Two-way ANOVA with Tukey's multiple comparisons test. *P < 0.05, **P < 0.01, ***P < 0.001. For (C) and (I), #P < 0.05, where the statistical significance was determined by unpaired t-test.


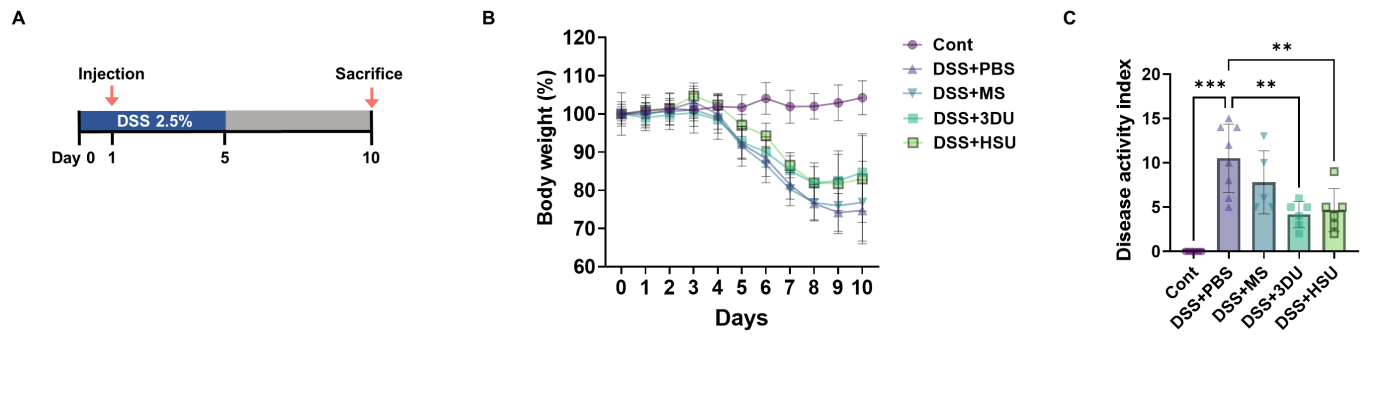


**Figure S6. Administration of 3D-MSC and Mel-HS confers prevention against DSS-induced colitis.** (A) Timeline for DSS administration and injection experiments. (B) Monitoring results for body weight changes for 10 days. (C) A dot plot displaying disease activity index (DAI) scores calculated on day 10.


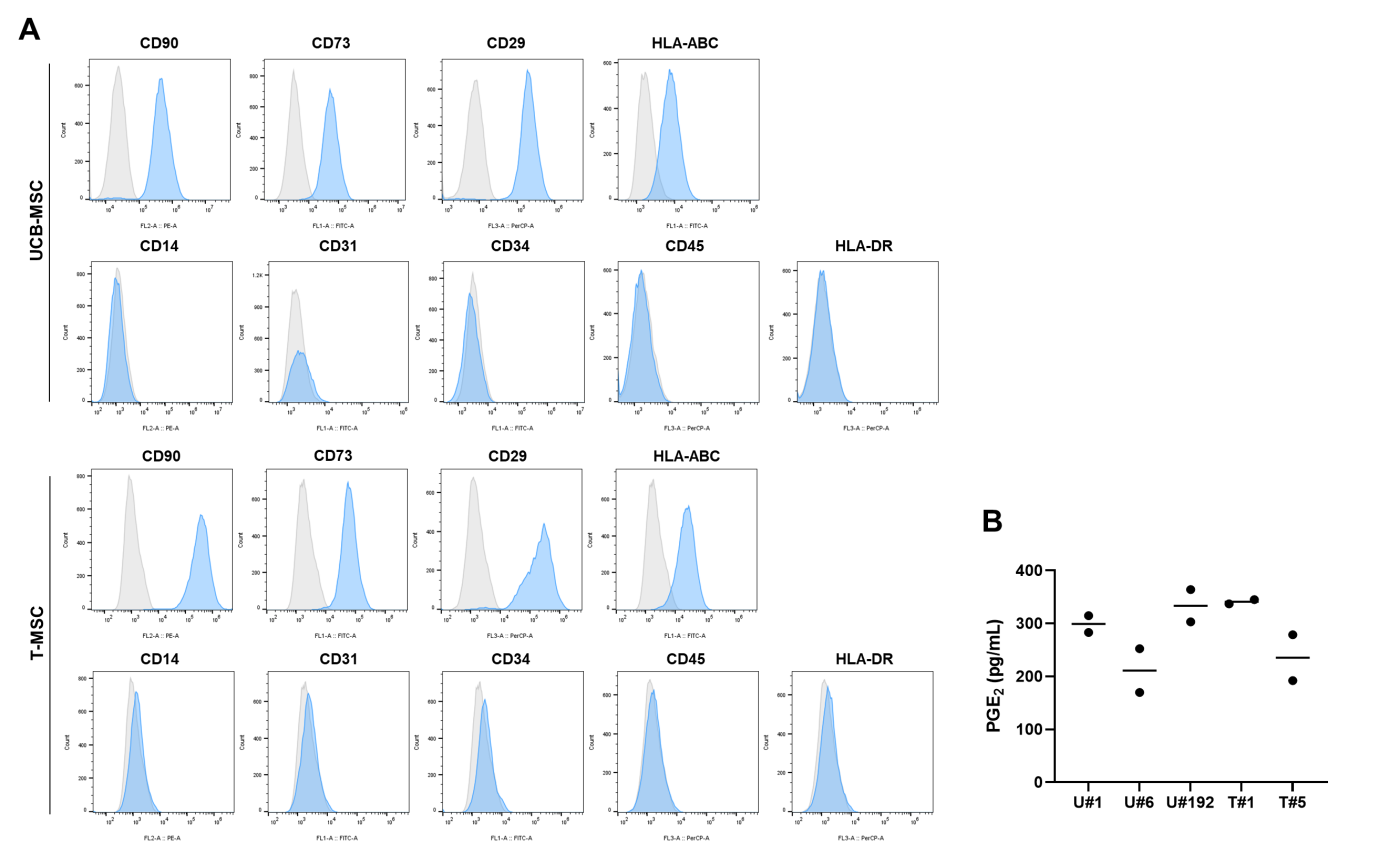


**Figure S7. MSC validation for Mel-HS generation.** Both UCB-MSCs and T-MSCs meet the requisite standards for MSC surface markers (A) and functional PGE2 secretion (B).

**
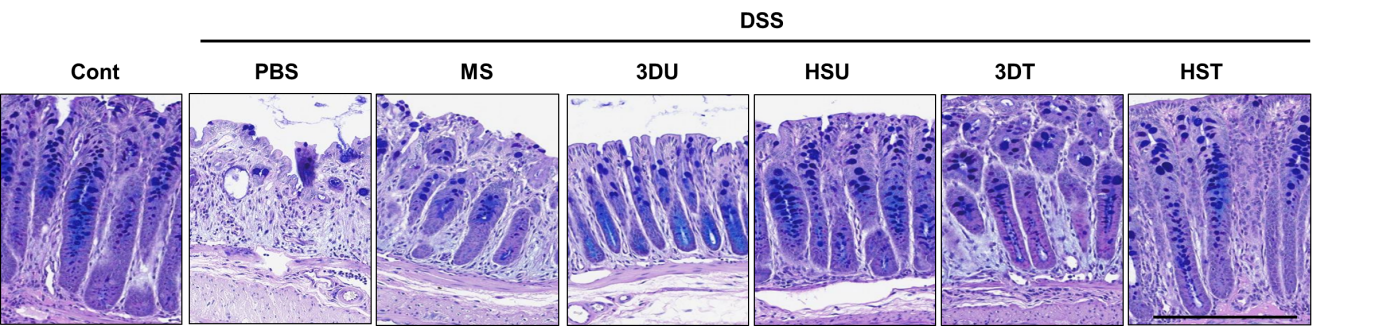
**

**Figure S8. Administration of 3D-MSC and Mel-HS reduced the loss of Mucin-producing cells.** Magnified representative images for AB-PAS-stained colon sections from various treatment groups, related to Fig.5J.

**
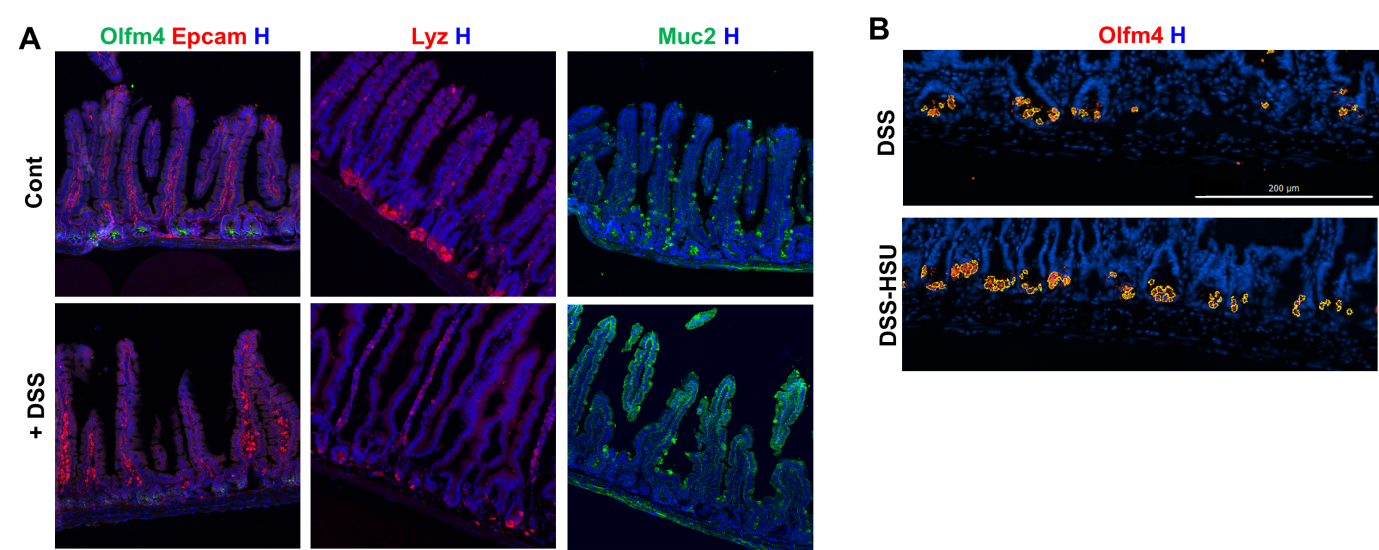
**

**Figure S9. Administration of DSS impairs mouse ileum.** (A) Representative immunohistochemical images of the ileum displaying specific marker expression. A significant loss of CBCs, Paneth cells and Goblet cells is evident following DSS treatment. (B) Example of a processed image for the quantification of olfm4, related to Fig.6G.
